# Impact of Nutritional Counselling and Support on Body Mass Index Recovery and Treatment Outcomes Among Tuberculosis Patients in the Lao People’s Democratic Republic

**DOI:** 10.3390/tropicalmed10070198

**Published:** 2025-07-15

**Authors:** Donekham Inthavong, Hend Elsayed, Phonesavanh Keonakhone, Vilath Seevisay, Somdeth Souksanh, Sakhone Suthepmany, Misouk Chanthavong, Xaysomvang Keodavong, Phonesavanh Kommanivanh, Phitsada Siphanthong, Phengsy Sengmany, Buahome Sisounon, Jacques Sebert, Manami Yanagawa, Fukushi Morishita, Nobuyuki Nishikiori, Takuya Yamanaka

**Affiliations:** 1 National Tuberculosis Control Centre, Vientiane 01160, Laos; ndonekham@gmail.com (D.I.); sakhone_sntc@yahoo.com (S.S.); chanthavongeung@hotmail.com (M.C.); xaysomvangkeodavong@gmail.com (X.K.); phonesavanh_33@hotmail.com (P.K.); phitsada@yahoo.fr (P.S.); sebertj5@gmail.com (J.S.); 2 Integrated Communicable Disease Control, World Health Organization Regional Office for the Western Pacific, Manila 1000, Philippines; hend.elabbasy@hotmail.com; 3 National Nutrition Centre, Vientiane 01160, Laos; phonesavanhkeonakhone64@gmail.com (P.K.); sengmanyphengsy99@gmail.com (P.S.); buahom.ssn@gmail.com (B.S.); 4 World Health Organization Country Office for Lao People’s Democratic Republic, Vientiane 01030, Laos; seevisayv@who.int (V.S.); souksanhs@who.int (S.S.); 5 The Research Institute of Tuberculosis, Japan Anti-Tuberculosis Association, Tokyo 204-8533, Japan; yanagawa_manami@jata.or.jp; 6 Global Tuberculosis Programme, World Health Organization, 1211 Geneva, Switzerland; nobu.nishikiori@gmail.com (N.N.); yamanakat@who.int (T.Y.)

**Keywords:** tuberculosis, undernutrition, body mass index, risk factors, treatment outcomes

## Abstract

Tuberculosis (TB) and undernutrition are intricately linked, significantly impacting health outcomes. However, nutritional support for TB patients is not systematically implemented in Lao People’s Democratic Republic (Lao PDR). This study evaluated the effects of nutritional counselling and support on nutritional recovery and TB treatment outcomes. A longitudinal study involved 297 individuals with drug-susceptible TB, 39.4% of whom had a body mass index (BMI) below 18.5 kg/m^2^. Participants were divided into an observation group and an intervention group, the latter receiving nutritional support. Nutritional support included ready-to-use therapeutic food and therapeutic milk products, tailored to patients’ nutritional status. Data collection was conducted at four intervals during treatment. By the end of treatment, 84.3% of participants improved their nutritional status to a BMI of 18.5 kg/m^2^ or higher. The intervention group showed early nutritional recovery, particularly during the intensive phase of TB treatment, although the *p*-value (*p* = 0.067) should be interpreted with caution. The overall treatment success rate was high at 90.6%, with no significant difference between groups. Factors associated with treatment success included age under 45, HIV-negative status, a BMI of 18.5 kg/m^2^ or higher, and clinically diagnosed pulmonary TB. Further assessment is required for the operational feasibility to provide systematic nutritional assessment and counselling for people with TB in Lao PDR.

## 1. Introduction

Tuberculosis (TB) is a cause of mortality, long-term disability and poverty, and it continues to be a global public health issue [1]. The World Health Organization (WHO) estimated that 10.8 million people newly fell ill with TB in 2023, and reported the highest number of TB case diagnosed (8.2 million) since WHO began global TB monitoring in 1995 [2]. This increase in case notifications is largely attributed to intensified case-finding efforts by national TB programs in the post-COVID-19 period. Although estimated TB mortality continued to decrease after the COVID-19 pandemic, TB became once again the leading cause of death from a single infectious agent in 2023, surpassing COVID-19 [2,3,4,5].

Undernutrition is frequently observed in people with TB and is recognized as a significant risk factor for active TB disease [6]. The relationship between TB and undernutrition is complex and bidirectional [7,8]. Undernutrition leads to an increased risk of developing active TB, with evidence suggesting that each unit increase body mass index (BMI) corresponds to a 13.8% reduction in the incidence of active TB [9]. Conversely, TB can contribute to undernutrition, as people with TB often suffer from reduced appetite and weight loss due to the metabolic changes associated with TB treatment [9,10,11,12,13]. A recent Cochrane review showed that undernutrition likely increases the risk of TB two-fold [14]. Recognizing the critical role of nutritional management in TB care, the WHO recommends that all people with TB receive nutritional assessment and receive appropriate nutritional counselling and care when needed, in line with general standards for the management of undernutrition [15,16].

Undernutrition not only increases the risk of active TB disease but also magnifies the severity of the disease and worsens the treatment outcomes, including increasing mortality rates [13,17,18,19]. The impact of undernutrition on TB treatment outcomes is particularly concerning. Studies have consistently shown that people with concurrent TB and undernutrition face higher risks of unfavorable TB treatment outcomes, including death [20,21,22,23]. This association is particularly pronounced in the context of drug-resistant TB (DR-TB) [10,24].

In Lao People’s Democratic Republic (Lao PDR), despite a steady annual decline in TB incidence since 2000, the rate remained high in 2023 at 132 cases per 100,000 population, surpassing both the WHO Western Pacific Region average and the global estimate [2,3]. TB treatment coverage (notified cases divided by estimated incidence) improved markedly from 37% in 2015 to 90% in 2023, driven by the scale-up of rapid molecular testing at diagnosis and active case finding in high-risk populations. However, TB prevalence among people screened remains high in certain localities, particularly in closed settings and remote areas with limited access to health services.

The country’s National Plan of Action on Nutrition 2021–2025 includes strategic objectives to prevent TB-related undernutrition [25]. However, systematic nutritional assessments and counselling are not routinely provided to people with TB, partly due to absence of national guidelines. Food support, previously offered as small daily incentives for TB patients, was discontinued over a decade ago due to limited resources. In addition, routine TB surveillance does not capture comprehensive data on nutritional status, such as weight, height and BMI. This study aimed to assess the impact of nutritional counselling and support on the recovery of nutritional status during TB treatment and on TB treatment outcomes.

## 2. Materials and Methods

### 2.1. Study Setting

Lao PDR is a lower-middle-income country with a population of approximately 7.7 million, the majority of whom reside in rural areas. The country has made significant strides in socio-economic development, particularly in poverty reduction and access to basic services. However, undernutrition remains a major public health concern, with 5.4% of the population undernourished [26], 24% of children under five classified as underweight and one-third experiencing stunting [27,28]. National nutrition priorities in Lao PDR are outlined in the National Socio-Economic Development Plan and the health sector reform strategy to achieve Universal Health Coverage [29,30]. These frameworks emphasize improving access to quality health and nutrition services for vulnerable populations, including through outreach services that integrate TB, HIV, and maternal and child health care.

Lao PDR also bears a high burden of TB with an incidence of 132 per 100,000 in 2023 [2]. TB diagnosis and treatment are provided for free of charge by the Lao National TB Programme (NTP) and the national health insurance (NHI) scheme. Social support equivalent to approximately USD 5 per day is provided by the NTP only for people with DR-TB [31]. In 2018–2019, Lao NTP conducted a national survey of costs incurred by people with TB and their households (national TB household cost survey) using the WHO recommended methods [32]. The survey recommended a wide range of policy interventions to minimize costs incurred by TB affected households. One of the recommendations is to improve nutritional support for TB patients including systematic nutrition assessment, counselling, and therapeutic and supplementary feeding for those in need, in coordination with the national nutrition centre and in line with the national nutrition strategy [31].

### 2.2. Study Design

This study was nested in a larger study assessing the impact of nutritional counselling and support on TB treatment outcomes and the financial burden due to TB. The description of the intervention is available elsewhere [33,34]. As a secondary objective, this study aimed to evaluate the effect of nutritional counselling and support on the early recovery of BMI during TB treatment, as well as its impact on TB treatment outcomes.

The study was conducted in six central and provincial hospitals, purposively selected based on operational feasibility and high TB case notification volumes (Supplementary Text S1). To account for site-wise differences in TB caseload, the sample size was allocated using a probability proportional to size (PPS) approach, based on the total number of notified TB cases in 2022. A total of 312 people with TB were enrolled in the larger study, corresponding to the planned sample size required to achieve 80% power to detect a minimum of 12.6% reduction in the proportion of TB-affected households facing catastrophic total costs due to TB, as estimated from the results of the national TB patient cost survey [31,35]. For this study, only people with drug-susceptible TB was included in the analysis.

The nutritional interventions of this study were provided by trained dieticians who were hired by the national nutrition programme of the Lao Ministry of Health. Eligible patients received either therapeutic milk products (F-75 and F-100) or ready-to-use therapeutic food (RUTF) such as Plumpy’Nut according to their clinical assessment. Study participants with very severe undernutrition (BMI ≤ 16.5 in adults or the mid-upper arm circumference (MUAC) < 11.5 cm in children) who had medical complication and/or no/poor appetite were provided with therapeutic milk products (F-75 and F-100) based on national guidelines on Integrated Management of Acute Malnutrition (IMAM) until they could receive RUTF. RUTF was provided from the start for participants who had a good appetite with severe malnutrition (BMI ≤ 16.5 in adults or MUAC < 11.5 cm in children) until they recovered to the level of BMI ≥ 16.5 in adults or MUAC ≥ 11.5 cm and < 12.5 cm in children. The necessary amount was calculated based on daily calorie intake per patient’s weight—40 kcal/kg/day. Given one package of RUTF has 500 kcal/package, for a patient with body weight of 60 kg, the necessary amount was four packages (fraction rounded down). For the patients getting to moderate malnutrition level (16.5 < BMI ≤ 18.5 in adult or MUAC ≥ 11.5 cm and < 12.5 cm in children), we maintained provision of micronutrients as a supplement or one package of Plumpy’Nut per day until recovery of BMI to the level of ≥ 18.5 in adults or MUAC ≥ 12.5 cm in children, after which we only provided nutritional monitoring and counselling. Nutritional supplements were distributed during routine drug pick-up or DOTS visits. Clinical dieticians provided repeated counselling to reinforce adherence and explain the role of supplementation in recovery. For the observation group, only standard TB care was provided, including general health education. Additional information on the intervention is provided in Supplementary Text S2.

### 2.3. Data Collection

Data collection items include (1) demographic information, e.g., age, sex, education, marital status, household size, employment status, and insurance status; (2) clinical information, e.g., mode of TB diagnosis, previous TB history, HIV status, smoking/alcohol behavior, drug use, and other comorbidities; (3) nutritional assessment, e.g., weight, height, BMI, and current appetite; and (4) TB treatment outcome at four time points, at approximately every two months during treatment. Anthropometric data were measured using standardized weight and height scales. We collected the data of BMI, self-reported weight and appetite changes at four standardized time points: (1) at time of TB diagnosis and starting TB treatment, (2) at the end of the TB treatment intensive phase, (3) during the middle, and (4) at the end of the TB treatment continuation phase. These follow-up assessments were conducted during routine TB care visits and were identical in timing and frequency for both the observation and intervention groups.

Clinical dieticians assigned at each study site served as interviewers for participant recruitment. Prior to being deployed to each study site, clinical dieticians received a 5-day training course on the study objectives, methods, and data collection tools. In addition to this, we conducted a 5-day pilot data collection to familiarize them with the process. During participants’ enrolment, clinical dieticians explained the purpose of the study and shared a written information sheet, in relevant local languages. Those who agreed to participate in the research and signed the informed consent form were enrolled. The data collection was conducted via in-person interviews at study sites, and the interview time was around 30–45 min.

This study used a non-randomized before-and-after design. Participants enrolled during the first three months were assigned to the observation group, and those enrolled during the subsequent three months were assigned to the intervention group. This sequential enrolment approach was used to reduce selection bias. Data were collected and entered at the time of interviews using tablet-based questionnaires with Ona and Open Data Kit (ODK) collect. Participant enrolment and data collection commenced on 10 January 2023, and all follow-up interviews were completed by 25 January 2024.

### 2.4. Data Analysis

Data cleaning and processing, statistical analyses, and data visualizations were performed using R4.4.1 software (CRAN: Comprehensive R Archive Network). For continuous data, descriptive statistics included mean with standard deviation (SD) and 95% confidence interval (CI), and median with interquartile range (IQR). Categorical data were presented as frequencies with proportion (%). A cut-off of BMI 18.5 kg/m^2^ was used to define undernutrition at the time of TB diagnosis (BMI < 18.5 kg/m^2^ categorized as undernutrition) [36,37]. Statistical differences between people with and without a low BMI were tested using a chi-square test for categorical data and either the t-test or Kruskal–Wallis test for continuous data. Statistical significance was defined as a *p*-value less than 0.05. Additionally, to assess the impact of the nutritional intervention on BMI recovery over the course of TB treatment, we fitted a linear mixed-effects model with BMI as the outcome, and treatment phase, study group (intervention vs. observation), and their interaction as fixed effects. Patient-level random intercepts were included to account for repeated BMI measures. Participants were included in the analysis up to the point of death, loss to follow-up, or withdrawal. Data collected prior to these events were retained, and no imputation was performed for missing values. Univariate logistic regression analysis was conducted to identify variables associated with undernutrition. Multivariate backward stepwise logistic regression was performed to identify the best model based on the Akaike information criterion. The selected final model was used for multivariate logistic regression analysis to calculate AOR and 95% CI.

### 2.5. Ethical Considerations

A written consent form was obtained from each participant prior to enrolment, explicitly stating that only the principal investigator (PI) and co-PIs were able to access the study dataset. Prior to obtaining a written informed consent, data collectors explained the purpose of this research with a written information sheet at each study site. Each participant’s voluntary will to continue participating in this study was also asked and confirmed at each data collection. Ethics approvals were also obtained from the Lao PDR National Ethics Committee for Health Research (Ref: 021/NECHR) and the Ethics Review Committee of the WHO Regional Office for the Western Pacific (Ref: 2022.3.LAO.1.ETB). Additionally, we obtained permission/endorsement from NTP and the department of disease control of Lao PDR Ministry of Health as well as from the hospital director of each study site, to conduct the data collection.

## 3. Results

**Figure 1:**
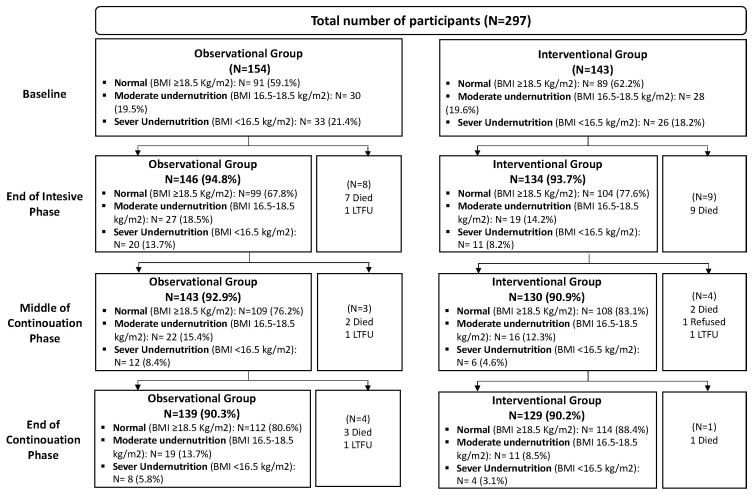
Study participant flowchart at each timepoint.

A total of 297 participants were enrolled in the study, with 154 in the observational group and 143 in the interventional group. Reasons for not completing four data collections included death (N = 24), loss-to-follow-up (N = 4) and refusal to continue the study participation (N = 1) (Figure 1).

### 3.1. Demographic and Clinical Characteristics

The mean age of participants was 48.3 years, and 10.1% had no formal education. Overall, 54.5% were covered by NHI and 36.4% reported no insurance (Table 1). Employment status showed a statistically significant difference between groups, as more participants in the intervention group were engaged in informal paid work compared to the observation group (51.7%) (*p* = 0.038). Half of the participants had smoking experience (40.1% were ex-smokers and 9.8% were current smokers), and the majority (71.4%) reported rarely or never consuming alcohol.

The majority of the TB cases (71.4%) were pulmonary TB with bacteriological confirmation. HIV status was predominantly negative (85.9%). Half of all participants experienced a diagnostic delay of more than 4 weeks from the onset of TB symptoms. This delay was slightly more prevalent in the observation group (53.2%) compared to the intervention group (44.8%), although the difference was not statistically significant (*p* = 0.178). Further comparisons by the status of completion of the four data collections are available in Supplementary Table S1. Data obtained from those who completed all four data collections (N = 268) were used as the basis for the analysis of the prevalence of undernutrition to ensure consistency in longitudinal comparisons.

### 3.2. Changes in BMI During TB Treatment

**Figure 2:**
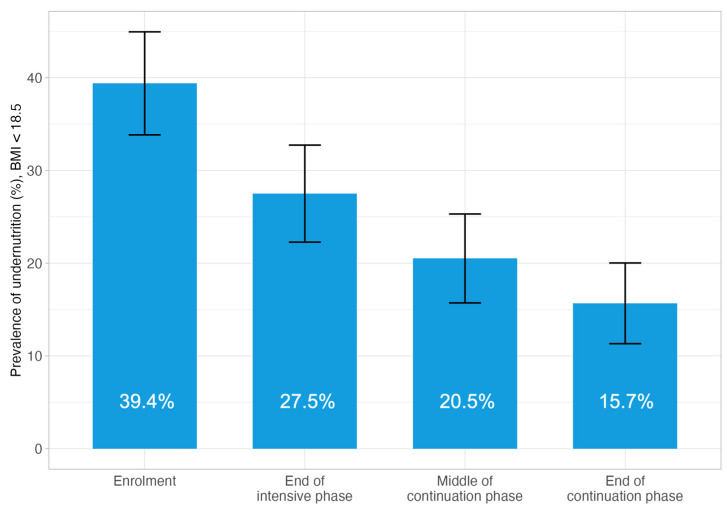
Prevalence of BMI < 18.5 at four time points during TB treatment, participants completed four data collections (N = 268). Error bars represent the 95% confidence interval.

At the TB diagnosis, the mean BMI for all participants was 19.7 (SD 3.6), and it recovered to 20.5 (SD 3.5) at the end of the TB intensive phase, 21.2 (SD 3.3) and 21.7 (SD 3.3) at the midpoint and endpoint of the TB continuation phase (Supplementary Table S2). Overall, the proportion of the study participants with BMI < 18.5 was 39.4%, and in line with the improvement in the mean BMI, the proportion reduced to 27.5% at the end of the TB intensive phase, and further to 20.5% and 15.7% at the midpoint and endpoint of the TB continuation phase (Figure 2).

**Figure 3:**
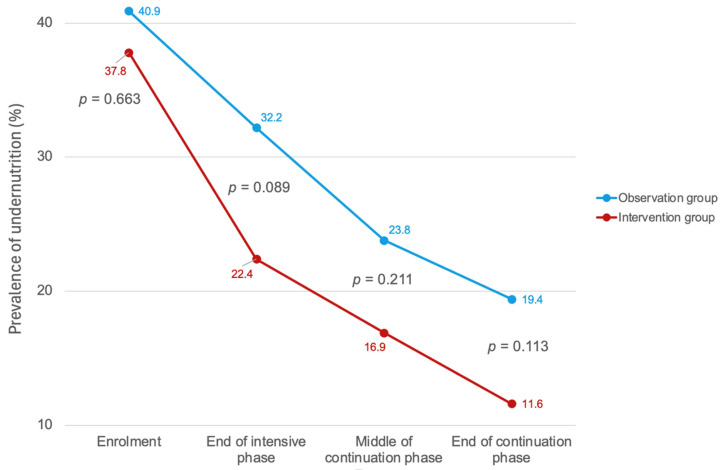
Prevalence of BMI < 18.5 at four time points during TB treatment, observation group vs. intervention group. Error bars represent the 95% confidence interval.

Comparing the proportion of the study participants with BMI < 18.5 in two groups, at the TB diagnosis, there was no significant difference between groups (40.9% in the observation group vs. 37.8% in the intervention group, *p* = 0.663) (Figure 3 and Table 2). At the end of the TB intensive phase, the intervention group showed a greater reduction in the proportion with BMI < 18.5, dropping to 22.4%, compared to 32.2% in the observation group (*p* = 0.089). This trend continued toward the end of the continuation phase; the proportion further decreased to 19.4% in the observation group and 11.6% in the intervention group, again with no significant difference observed (*p* = 0.113) (Figure 3 and Table 2).

The linear mixed-effects model showed a significant increase in BMI over time in both study groups. However, there was no statistically significant difference in BMI between the intervention and observation groups at any time point, as indicated by the non-significant group main effect (*p* = 0.51) and non-significant interaction terms (*p* > 0.1 for all time points). (Supplementary Table S3).

### 3.3. TB Treatment Outcomes

The proportion of participants achieving treatment success was high at 90.6%, with no significant difference between two groups, with 139 (90.3%) in the observation group and 130 (90.9%) in the intervention group (*p* = 0.639). Death occurred in 12 participants (7.8%) in the observation group and 12 participants (8.4%) in the intervention group, representing an overall death rate of 8.1% (N = 24). Similarly, the number of participants lost to follow-up (LTFU) was low, with three (1.9%) in the observation group and one (0.6%) in the intervention group, for a combined LTFU rate of 1.3% (Table 3).

A univariate logistic regression showed that providing nutritional counselling and support was not associated with TB treatment success (OR = 1.08, *p* = 0.848). Multivariate logistic regression identified other factors associated with a higher TB treatment success rate: age group 0–44 years compared to 65 years or more (AOR = 4.26, *p* = 0.031), HIV-negative compared to HIV-positive (AOR = 9.49, 95% CI: 2.83–34.67, *p* < 0.001), clinically diagnosed pulmonary TB to bacteriologically confirmed cases (AOR = 3.26, *p* = 0.060), female compared to male (AOR = 2.30, *p* = 0.094) and BMI ≥ 18.5 at TB diagnosis compared to BMI <18.5 (AOR = 1.85, *p* = 0.153) (Table 4). Factors associated with deaths due to TB were presented in Supplementary Table S4.

## 4. Discussion

Undernutrition is known as a risk factor of active TB disease and also of unfavorable TB treatment outcomes, including increased TB mortality [13,17,18,19]. This study observed a trend suggesting that the nutritional counselling and support may have contributed to early BMI recovery among people with TB. At enrolment, 39.4% of the participants were classified as undernourished, which decreased to 27.5% by the end of the intensive treatment phase. This trend continued through the middle of the continuation phase, where the prevalence of undernutrition further declined to 20.5%, and reached its lowest point at the end of the continuation phase at 15.7%. These results indicate a progressive improvement in nutritional status over the course of TB treatment. The most notable reduction in undernutrition occurred between enrolment and the intensive phase. However, this did not translate into significant differences in TB treatment outcomes, due to the limitation of study sample size.

A recent meta-analysis revealed that people with TB and undernutrition face an increased likelihood of unfavorable treatment outcomes (AOR = 1.7, 95% CI: 1.4–1.9), a higher risk of mortality (AOR = 3.1, 95% CI: 2.4–3.9), and an elevated chance of treatment failure or recurrence (AOR = 1.6, 95% CI: 1.2–2.0) compared to those with adequate nutritional status [38]. A retrospective cohort study conducted in Taiwan indicated that undernutrition was a significant predictor of all causes of TB mortality (AOR = 2.22, 95% CI, 1.45–3.40) [39]. These findings from previous studies underscore the potential importance of early BMI improvement in enhancing TB treatment outcomes.

Another systematic review found that nutritional support was associated with improved treatment adherence in five out of eight studies which reflect positively on treatment outcomes [40]. A retrospective cohort study in India demonstrated significantly improved treatment outcomes in people with TB who received nutritional supplementation. The cure rate was substantially higher in the intervention group (74.7% vs. 50.9%) (OR = 2.86, 95% CI: 2.26–3.61, *p* < 0.001), and mortality was significantly reduced (1.1% vs. 5.7%) (OR = 0.18, 95% CI: 0.08–0.41, *p* < 0.001) [41]. Similarly, a large cohort study in India demonstrated that patients who gained at least 5% of their baseline weight in the first 2 months of treatment had a 61% reduced hazard of death (adjusted hazard ratio 0.39, 95% CI: 0.18–0.86) [10]. Although this study was not able to detect a significant difference in TB treatment outcomes between the observation and intervention groups, the observed trend of early BMI recovery and supporting evidence from other settings underscore the potential value of integrating nutritional support into TB care.

To further explore this finding, we examined subgroup characteristics and potential confounding factors. Although the prevalence of undernutrition (BMI < 18.5 kg/m^2^) at TB diagnosis was slightly lower in the intervention group (37.8% vs. 40.9%), the death rate among participants with undernutrition was higher (14.8% vs. 9.4%). The intervention group also had a slightly higher proportion of individuals with known risk factors for TB mortality, including males (67.8% vs. 57.1%), HIV-positive status (15.4% vs. 12.3%), and current or former smokers (53.9% vs. 48.1%). Notably, half of the deaths in the intervention group (N = 6/12) occurred among HIV-positive individuals, compared to 25% (N = 3/12) in the observation group. These differences may reflect the influence of limited sample size and sequential enrollment, which can increase variability and lead to imbalances in baseline characteristics. Such confounding factors may have attenuated the observed impact of nutritional support on treatment success.

This study contributes to addressing a critical knowledge gap in Lao PDR by exploring the potential effects of nutritional interventions on TB treatment outcomes. Based on the observed trends and existing evidence from other settings, it may be beneficial for the Lao NTP to consider piloting systematic nutritional assessments for people diagnosed with TB and exploring options for integrating this data into the digital case-based surveillance system. While the study showed promising trends in nutritional improvement, especially during the intensive phase, future research with larger sample sizes and longer follow-up periods is needed to measure the impact of nutritional interventions on TB treatment outcomes. Meanwhile, continued monitoring of TB treatment outcomes, particularly among older adults, people living with HIV, and people with low BMI at diagnosis, through the real-time case-based TB information system (TB tracker) remains an important component of programmatic surveillance.

Our study found that younger individuals (<45 years) had significantly higher treatment success rates compared to older adults (≥65 years) (AOR = 4.26, 95% CI: 1.17–17.1, *p* = 0.031). This aligns with findings from a study done in eastern Ethiopia, which reported that patients aged 46–54 years had 10.4 times higher odds of unsuccessful treatment outcomes compared to younger people (AOR = 10.41, 95% CI: 1.86–58.30) [42]. Another study in Zambia found that patients aged ≥ 65 years had 72.4% lower odds of treatment success compared to those aged 15–24 years (OR = 0.276, 95% CI: 0.086–0.881, *p* = 0.030) [43]. A meta-analysis found that patients younger than 65 years were twice as likely to succeed in treatment (OR = 2.0, 95% CI: 1.7–2.4) [44]. A study in Lesotho showed that the odds of successful TB treatment outcome were higher for the 20–24 years age group (88.2% vs. 65.3%, OR = 3.98, 95% CI: 1.42–11.22, *p* = 0.009) and 55–59 years (91.7% vs. 65.3%, OR = 5.84, 95% CI: 1.56–21.88, *p* = 0.009), compared to ≥ 65 years age group [45]. The consistency across these studies highlights age as a key factor influencing TB treatment outcomes. Younger individuals may achieve higher success rates due to factors such as stronger immune systems [46], fewer comorbidities [47], better adherence to treatment, and greater tolerance to medication side effects [46].

The strong association between HIV-negative status and treatment success (AOR = 9.49, 95% CI: 2.83–34.67, *p* < 0.001) in our study is supported by a study in Thailand reported that people with TB-HIV-positive had 3.1 times higher risk of unsuccessful treatment outcomes compared to HIV-negative patients [48]. A study in South Africa found that HIV-negative individuals had nearly five times greater odds of having a successful TB treatment outcome compared to HIV-positive (OR = 4.98, 95% CI: 2.07–11.25) [49]. This trend is further corroborated by a study in Addis Ababa, Ethiopia, which found that HIV-positive TB patients had 2.7 times higher odds of unsuccessful treatment outcomes [42]. The consistency of these findings across different geographical locations underscores the need for differentiated care models tailored to the specific needs of TB–HIV co-infected individuals. Integrated TB–HIV services, enhanced clinical monitoring, and targeted nutritional and psychosocial support may be critical to improving outcomes in this high-risk population.

Although not statistically significant, our study observed a trend suggesting that people with higher BMI at the time of TB diagnosis may experience better treatment outcomes (92.8% vs. 87.2%, AOR = 1.85, *p* = 0.153). This trend aligns with findings from a study in Ethiopia, which demonstrated that patients with a BMI ≥ 18.5 kg/m^2^ at treatment initiation had 2.15 times higher odds of treatment success (AOR = 2.15, 95% CI: 1.05–4.39), with a success probability of 92.9% compared to 86.5% for those with a BMI < 18.5 kg/m^2^ [40]. Similarly, a study in South Korea found that normal or overweight patients had three times higher odds of successful treatment outcomes versus those who were underweight (OR = 0.33, 95% CI:  0.20–0.54) [50]. Patients with higher BMIs may benefit from a stronger immune function [51], improved drug metabolism [40], and greater physical strength [38], contributing to more favorable TB treatment outcomes.

Although smoking and alcohol use are known to influence TB treatment outcomes [52,53], our study did not find statistically significant associations between these behaviors and treatment success. The prevalence of current smoking in our sample was slightly lower than national estimates, which may reflect behavior change following TB symptom onset or diagnosis. While we believe underreporting was minimized through careful questionnaire design and interviewer training, some degree of social desirability bias cannot be ruled out. Additionally, the lack of statistical significance may be due to the limited sample size in this study. TB treatment outcomes are influenced by a complex interplay of factors, including medication adherence, socioeconomic conditions, and comorbidities, which may have confounded or masked the effect of the nutritional intervention.

This study has several limitations. First, the limited sample size and the suboptimal intensity and design of the intervention may have constrained the ability to detect statistically significant differences in BMI and TB treatment outcomes between the intervention and observation groups. The current analysis focuses on a secondary objective of the broader study; therefore, the sample size was not specifically powered for comparisons related to nutritional recovery or treatment outcomes. Additionally, approximately 60% of participants in both study groups had a BMI ≥ 18.5 kg/m^2^ at enrolment and thus did not receive supplementary or therapeutic feeding. This likely diluted the measurable impact of the intervention. We also acknowledge that some participants in the observation group may have received informal nutritional advice from data collectors who were trained clinical dieticians. This was not part of the planned intervention but may have occurred naturally during interactions at the study sites. Such instances could have contributed to a narrowing of the difference between the two groups. These findings suggest the need for future studies with larger sample sizes and more intensive or personalized nutritional support strategies, particularly targeting individuals with moderate or severe undernutrition. In addition, identifying and promoting the use of nutritious, locally available foods could enhance the sustainability and cultural acceptability of nutritional interventions, particularly in rural or resource-constrained settings.

Second, the study was conducted in purposively selected central and provincial hospitals, which limits the generalizability of the findings. Health facilities in rural areas where undernutrition is often more prevalent [54,55] were not included. A nationwide intervention study would be required to comprehensively assess the association between nutritional support and TB treatment outcomes across diverse settings. Scaling up such an initiative would require strategic discussions at the national and provincial levels on the integration of a monitoring mechanism of nutritional status of people with TB into routine TB surveillance systems.

Third, this study did not include people with DR-TB due to the limited number of cases notified in the country. Since people with DR-TB are more likely to experience undernutrition and have lower TB treatment success rates, future national and subnational policies addressing TB-related undernutrition should explicitly include this population [33,56].

## 5. Conclusions

This study was the first in Lao PDR to assess the impact of nutritional counselling and support on early recovery of BMI during TB treatment and on TB treatment success rate. We observed a trend suggesting that the nutritional counselling and support may have contributed to early BMI recovery, particularly during the intensive phase of TB treatment. However, this did not translate into statistically significant differences in TB treatment outcomes, possibly due to limitations in sample size and intervention intensity. While these findings do not yet provide definitive evidence to support nationwide scale-up, they offer important operational insights and highlight the potential value of integrating nutrition into TB care. Further assessment is required to confirm the impact of such interventions on clinical outcomes at scale and to evaluate their operational feasibility and scalability in the Lao PDR context.

## Figures and Tables

**Table 1:** Socio-demographic, and TB-related characteristics by study group.

Variable	Category	Observation Group N (%)	Intervention Group N (%)	Total N (%)	*p*-Value
Total		154 (51.9)	143 (48.1)	297 (100)	-
Demographic characteristics					
Age group (years)	Mean (SD)	48.9 (18.3)	47.7 (16.1)	48.3 (17.3)	0.567
	0–14	1 (0.6)	0 (0.0)	1 (0.3)	0.347
	15–24	16 (10.4)	11 (7.7)	27 (9.1)	
	25–34	28 (18.2)	25 (17.5)	53 (17.8)	
	35–44	17 (11.0)	23 (16.1)	40 (13.5)	
	45–54	26 (16.9)	30 (21.0)	56 (18.9)	
	55–64	28 (18.2)	31 (21.7)	59 (19.9)	
	65+	38 (24.7)	23 (16.1)	61 (20.5)	
Sex	Female	66 (42.9)	46 (32.2)	112 (37.7)	0.075
	Male	88 (57.1)	97 (67.8)	185 (62.3)	
Marital status	Single	40 (26.0)	32 (22.4)	72 (24.2)	0.289
	Married	87 (56.5)	93 (65.0)	180 (60.6)	
	Divorced/separated/widowed	27 (17.5)	18 (12.6)	45 (15.2)	
Insurance type	None	52 (33.8)	56 (39.2)	108 (36.4)	0.557
	National Health Insurance	86 (55.8)	76 (53.1)	162 (54.5)	
	Community-Based Health Insurance	2 (1.3)	1 (0.7)	3 (1.0)	
	Social Security Organization	5 (3.2)	4 (2.8)	9 (3.0)	
	State Authority for Social Security	6 (3.9)	5 (3.5)	11 (3.7)	
	Private health insurance	3 (1.9)	0 (0.0)	3 (1.0)	
	Other	0 (0.0)	1 (0.7)	1 (0.3)	
Smoker	No smoking experience	83 (53.9)	66 (46.2)	149 (50.2)	0.383
	Current smoker	13 (8.4)	16 (11.2)	29 (9.8)	
	Ex-smoker	58 (37.7)	61 (42.7)	119 (40.1)	
Alcohol use	Daily	10 (6.5)	15 (10.5)	25 (8.4)	0.324
	Weekly	13 (8.4)	18 (12.6)	31 (10.4)	
	Monthly	17 (11.0)	12 (8.4)	29 (9.8)	
	Rarely/Never	114 (74.0)	98 (68.5)	212 (71.4)	
Educational level	No education	20 (13.0)	10 (7.0)	30 (10.1)	0.249
	Primary	40 (26.0)	36 (25.2)	76 (25.6)	
	Lower/higher secondary	69 (44.8)	65 (45.5)	134 (45.1)	
	Diploma or higher, vocational, other	25 (16.2)	32 (22.4)	57 (19.2)	
Employment status before TB	Unemployed	44 (28.6)	31 (21.7)	75 (25.3)	0.038
	Formal paid work	19 (12.3)	27 (18.9)	46 (15.5)	
	Informal paid work	67 (43.5)	74 (51.7)	141 (47.5)	
	Retired/student/housework/other	24 (15.6)	11 (7.7)	35 (11.8)	
Clinical characteristics					
TB type	Pulmonary, bacteriologically confirmed	111 (72.1)	101 (70.6)	212 (71.4)	0.800
	Pulmonary, bacteriologically unconfirmed (clinically diagnosed)	36 (23.4)	33 (23.1)	69 (23.2)	
	Extrapulmonary	7 (4.5)	9 (6.3)	16 (5.4)	
HIV status	HIV negative	135 (87.7)	120 (83.9)	255 (85.9)	0.428
	HIV positive	19 (12.3)	22 (15.4)	41 (13.8)	
	HIV test not done	0 (0.0)	1 (0.7)	1 (0.3)	
Treatment history	New	145 (94.2)	136 (95.1)	281 (94.6)	0.227
	Relapse	6 (3.9)	7 (4.9)	13 (4.4)	
	Retreatment	3 (1.9)	0 (0.0)	3 (1.0)	
Diagnostic delay	No	72 (46.8)	79 (55.2)	151 (50.8)	0.178
	Yes (>4 weeks from onset of TB symptoms)	82 (53.2)	64 (44.8)	146 (49.2)	

**Table 2:** Prevalence and reduction of undernutrition and severe undernutrition over treatment phases.

Variables	Phase	Observation Group	Intervention Group	*p*-Value
Prevalence of BMI * < 18.5	At TB diagnosis	40.9%	37.8%	0.663
	End of intensive phase	32.2%	22.4%	0.089
	Middle of continuation phase	23.8%	16.9%	0.211
	End of continuation phase	19.4%	11.6%	0.113
Prevalence of BMI * < 16.5	At TB diagnosis	21.4%	18.2%	0.773
	End of intensive phase	13.7%	8.2%	0.164
	Middle of continuation phase	8.4%	4.6%	0.311
	End of continuation phase	5.8%	3.1%	0.211

* BMI: Body Mass Index.

**Table 3:** TB treatment outcomes by study group.

Variable	Category	Observation Group; N (%)	Intervention Group; N (%)	Total; N (%)	*p*-Value
Treatment outcomes	Treatment success	139 (90.3)	130 (90.9)	269 (90.6)	0.639
	Treatment failure	0 (0.0)	0 (0.0)	0 (0.0)	
	Died	12 (7.8)	12 (8.4)	24 (8.1)	
	Lost to follow-up	3 (1.9)	1 (0.6)	4 (1.3)	
Occurrence of deaths during TB treatment	End of intensive phase	7 (4.6)	9 (6.3)	16 (5.4)	0.929
	Middle of continuation phase	2 (1.3)	2 (1.4)	4 (1.4)	
	End of continuation phase	3 (1.9)	1 (0.7)	4 (1.4)	

**Table 4:** Factors determining TB treatment success.

Variable	Category	N	n (%)	*p*-Value	Crude OR (95% CI, *p*-Value)	Adjusted OR (95% CI, *p*-Value)
Age group	65+	61	54 (88.5%)	0.781	Ref	Ref
	0–44	121	111 (91.7%)		1.44 (0.50–3.95, *p* = 0.484)	4.26 (1.17–17.01, *p* = 0.031)
	45–64	115	104 (90.4%)		1.23 (0.43–3.29, *p* = 0.691)	1.44 (0.48–4.11, *p* = 0.504)
Sex	Male	185	163 (88.1%)	0.096	Ref	Ref
	Female	112	106 (94.6%)		2.38 (0.99–6.65, *p* = 0.069)	2.30 (0.92–6.62, *p* = 0.094)
Marital status	Single	72	65 (90.3%)	0.897	Ref	-
	Married	180	164 (91.1%)		1.10 (0.41–2.72, *p* = 0.836)	-
	Divorced/separated/widowed	45	40 (88.9%)		0.86 (0.26–3.08, *p* = 0.810)	-
Insurance type	No insurance	108	95 (88.0%)	0.339	Ref	-
	With insurance	189	174 (92.1%)		1.59 (0.72–3.48, *p* = 0.248)	-
Smoking status	Current smoker	29	23 (79.3%)	0.059	Ref	-
	No smoking experience	149	139 (93.3%)		3.63 (1.14–10.77, *p* = 0.022)	-
	Ex-smoker	119	107 (89.9%)		2.33 (0.75–6.67, *p* = 0.125)	-
Alcohol use	Daily	25	23 (92.0%)	0.580	Ref	-
	Weekly	31	26 (83.9%)		0.45 (0.06–2.32, *p* = 0.369)	-
	Monthly	29	27 (93.1%)		1.17 (0.13–10.42, *p* = 0.877)	-
	Rarely/Never	212	193 (91.0%)		0.88 (0.14–3.32, *p* = 0.873)	-
HIV status	HIV positive	41	32 (78.0%)	0.012	Ref	Ref
	HIV negative	255	236 (92.5%)		3.49 (1.40–8.22, *p* = 0.005)	9.49 (2.83–34.67, *p* < 0.001)
	Status unknown	1	1 (100.0%)		-	-
Educational level	No education	30	26 (86.7%)	0.717	Ref	-
	Primary	76	71 (93.4%)		2.18 (0.51–8.88, *p* = 0.270)	-
	Lower/higher secondary	134	121 (90.3%)		1.43 (0.38–4.43, *p* = 0.557)	-
	Diploma or higher, vocational, other	57	51 (89.5%)		1.31 (0.31–4.99, *p* = 0.697)	-
Employment status before TB	Unemployed	75	69 (92.0%)	0.820	Ref	-
	Formal paid work	46	40 (87.0%)		0.58 (0.17–1.97, *p* = 0.372)	-
	Informal paid work	141	128 (90.8%)		0.86 (0.29–2.27, *p* = 0.763)	-
	Retired/student/housework/other	35	32 (91.4%)		0.93 (0.23–4.61, *p* = 0.919)	-
Nutritional intervention	Without any intervention	154	139 (90.3%)	1.000	Ref	-
	With nutritional intervention	143	130 (90.9%)		1.08 (0.49–2.39, *p* = 0.848)	-
TB type	Pulmonary, bacteriologically confirmed	212	190 (89.6%)	0.480	Ref	Ref
	Pulmonary, clinically diagnosed	69	65 (94.2%)		1.88 (0.69–6.61, *p* = 0.261)	3.26 (1.05–12.87, *p* = 0.060)
	Extrapulmonary	16	14 (87.5%)		0.81 (0.21–5.38, *p* = 0.790)	1.17 (0.24–9.11, *p* = 0.859)
Treatment history	Relapse/retreatment	16	14 (87.5%)	1.000	Ref	-
	New	281	255 (90.7%)		1.40 (0.21–5.39, *p* = 0.667)	-
Household size	≥5	149	131 (87.9%)	0.170	Ref	Ref
	<5	148	138 (93.2%)		1.90 (0.86–4.41, *p* = 0.121)	2.21 (0.95–5.49, *p* = 0.073)
Diagnostic delay	No	151	136 (90.1%)	0.916	Ref	-
	Yes	146	133 (91.1%)		1.13 (0.52–2.50, *p* = 0.762)	-
BMI at TB diagnosis	<18.5	117	102 (87.2%)	0.159	Ref	Ref
	≥18.5	180	167 (92.8%)		1.89 (0.86–4.19, *p* = 0.111)	1.85 (0.79–4.37, *p* = 0.153)

Ref: reference category used for comparison when calculating odds ratios.

## Data Availability

The study dataset contains privacy-sensitive information including participants’ individual and household income that formed a core part of the analysis. Even though we removed patients’ identifiers such as patient number and name, there is still a possibility that those who are familiar with the project sites and beneficiaries may be able to identify participants and their households. The informed consent signed by all participants explicitly mentioned that only the research team have access to the dataset. Due to such ethical and confidentiality restrictions, the survey dataset will be made available only upon request and with permission from the National Tuberculosis Control Programme (NTP), Ministry of Health, Lao PDR. All interested researchers may contact the NTP of Lao PDR (ndonekham@gmail.com and phonesavanh_33@hotmail.com), and/or, for non-author contact, the WHO/WPRO ERC (wproethicsreviewcomm@wpro.who.int).

## Supplementary Materials

The following supporting information can be downloaded at https://www.mdpi.com/article/10.3390/tropicalmed10070198/s1, Text S1: Additional information on study design; Text S2: Additional information on the intervention; Table S1: Demographic and clinical characteristics of study participants, by status of the completion of data collection; Table S2: Body mass index at four-time points of data collection, by study group; Table S3: Results of linear mixed-effect model assessing BMI change over TB treatment phases, by study group; Table S4: Factors associated with deaths due to TB.
