# Supplementary material for: Impact of Nutritional Counselling and Support on Body Mass Index Recovery and Treatment Outcomes Among Tuberculosis Patients in the Lao People’s Democratic Republic
Source: Trop Med Infect Dis. 2025 Jul 15;10(7):198. doi: 10.3390/tropicalmed10070198 (PMC12300389; doi:10.3390/tropicalmed10070198)
Supplement: Supplementary file 1 [file tropicalmed-10-00198-s001.zip › tropicalmed-3646689-supplementary.pdf]

Supplementary information

Text S1. Additional information on study design

Study sites: Mittaphap hospital and Setthathirath hospital (Vientiane capital), Khammuan provincial hospital (Khammuan province), Luanprabang provincial hospital (Luanprabang province), Savannakhet provincial hospital (Savannakhet province) and Champasack provincial hospital (Champasack province).

Inclusion criteria: 1) participants (including children) who are newly diagnosed as drug-susceptible TB (DS-TB); 2) consent to the study participation within seven days of TB diagnosis; 3) including new, relapse and retreatment TB cases. Data collection was conducted four times over the course of the TB treatment.

Text S2. Additional information on the intervention

- Nutritional counselling was delivered by trained clinical dieticians using a standardized approach and materials. Counselling sessions included assessment of dietary intake and preferences, followed by tailored advice on improving the nutritional quality and caloric density of locally available foods. Dieticians discussed appropriate meal composition, frequency of meals and snacks, and strategies to enhance energy intake using accessible ingredients. Patients were advised on the use of supplements such as milk powder, groundnuts, and pulses, which were framed as essential therapeutic components to support recovery of lost weight and muscle mass. Family members were also engaged during counselling to reinforce the importance of ensuring that these supplements were consumed primarily by the patient.
- Counselling was provided at each of the four scheduled follow-up visits during TB treatment, with additional reinforcement during routine drug pick-up or DOTS visits.
- To ensure consistency across sites, all dieticians underwent centralized training that covered counselling content, anthropometric assessment, and feeding protocols. Supervision and periodic quality checks were conducted by national programme staff.
- Summary of nutritional support by BMI/MUAC category

BMI/MUAC Category	Clinical Condition	Nutritional Support Provided	Frequency/Duration

**BMI > 18.5 (adults) or MUAC ≥ 12.5 cm (children)**	Normal nutritional status	Provide information of daily calorie intake to sustain BMI level	At each follow-up visit
**16.5 < BMI ≤ 18.5 (adults) or MUAC 11.5–12.5 cm (children)**	Moderate malnutrition	Plumpy'Nut one package per day or micronutrients as a supplement	Until BMI ≥ 18.5 (adults) or MUAC ≥ 12.5 cm (children)
**BMI ≤ 16.5 (adults) or MUAC < 11.5 cm (children)**	Acute malnutrition with good appetite	Plumpy'Nut. Amount based on patient's body weight (see the table below)	Daily, until BMI recovers to > 16.5
	Acute malnutrition with poor appetite or medical complications	Therapeutic milk (F-75/F-100)	Until appetite improves and BMI/MUAC reaches next threshold

- Examples of necessary calorie intake and amount of RUTF packages based on body weight

**Body Weight**	**Necessary Calories per day**<br>(40 kcal/kg/day)	**Amount of RUTF**<br>(500 kcal/package, fraction rounded down)
**30**	1200 kcal	2 packages
**35**	1400 kcal	3 packages
**40**	1600 kcal	3 packages
**45**	1800 kcal	3 packages
**50**	2000 kcal	4 packages
**55**	2200 kcal	4 packages
**60**	2400 kcal	4 packages

Table S1. Demographic and clinical characteristics of study participants by data collection completion status

Variables		Complete		Drop out		All enrolment		p-value
		N	(%)	N	(%)	N	(%)	
**Total**		**268**	**90.2%**	**29**	**9.8%**	**297**	**100%**	
**Demographic characteristics**								
Age group	0–14	1	0.4%	0	0.0%	1	0.3%	0.865
	15–24	24	9.0%	3	10.3%	27	9.1%	
	25–34	50	18.7%	3	10.3%	53	17.8%	
	35–44	37	13.8%	3	10.3%	40	13.5%	
	45–54	51	19.0%	5	17.2%	56	18.9%	
	55–64	52	19.4%	7	24.1%	59	19.9%	
	≥65	53	19.8%	8	27.6%	61	20.5%	
Sex	Female	106	39.6%	6	20.7%	112	37.7%	0.074
	Male	162	60.4%	23	79.3%	185	62.3%	
Education level	No education	26	9.7%	4	13.8%	30	10.1%	0.856
	Primary	70	26.1%	6	20.7%	76	25.6%	
	Lower/higher secondary	121	45.1%	13	44.8%	134	45.1%	
	Diploma or higher, vocational, other	51	19.0%	6	20.7%	57	19.2%	
Insurance status	None	95	35.4%	13	44.8%	108	36.4%	0.654
	National Health Insurance (NHI) scheme	148	55.2%	14	48.3%	162	54.5%	
	Community-Based Health Insurance (CBHI)	2	0.7%	1	3.4%	3	1.0%	
	Health Equity Fund (HEF)	0	0.0%	0	0.0%	0	0.0%	
	Social Security Organization (SSO) for salaried private-sector employees	9	3.4%	0	0.0%	9	3.0%	
	State Authority for Social Security (SASS) for civil servants	10	3.7%	1	3.4%	11	3.7%	
	Private health insurance	3	1.1%	0	0.0%	3	1.0%	
	Other	1	0.4%	0	0.0%	1	0.3%	
Employment status before TB	Unemployed	67	25.0%	8	27.6%	75	25.3%	0.722
	Formal paid work	40	14.9%	6	20.7%	46	15.5%	
	Informal paid work	128	47.8%	13	44.8%	141	47.5%	
	Retired/student/housework/other	33	12.3%	2	6.9%	35	11.8%	
Household size	≥ 5	130	48.5%	19	65.5%	149	50.2%	0.122
	< 5	138	51.5%	10	34.5%	148	49.8%	
Primary income earner	No	162	60.4%	18	62.1%	180	60.6%	0.925
	Yes	91	34.0%	9	31.0%	100	33.7%	
	Equal contributor	15	5.6%	2	6.9%	17	5.7%	
**Clinical characteristics**								
TB type	Pulmonary, bacteriologically confirmed	189	70.5%	23	79.3%	212	71.4%	0.438
	Pulmonary, bacteriologically unconfirmed	65	24.3%	4	13.8%	69	23.2%	
	Extrapulmonary	14	5.2%	2	6.9%	16	5.4%	
Treatment history	New	254	94.8%	27	93.1%	281	94.6%	0.375
	Relapse	12	4.5%	1	3.4%	13	4.4%	
	Retreatment	2	0.7%	1	3.4%	3	1.0%	
HIV status	HIV positive	32	11.9%	9	31.0%	41	13.8%	0.018
	HIV negative	235	87.7%	20	69.0%	255	85.9%	
	Status unknown	1	0.4%	0	0.0%	1	0.3%	
Diagnostic delay* (> 4weeks)		133	49.6%	13	44.8%	146	49.2%	0.768
Body mass index at TB diagnosis	< 18.5	101	37.7%	16	55.2%	117	39.4%	0.103
	≥ 18.5	167	62.3%	13	44.8%	180	60.6%	
**Financial status**		**Mean**	**95%CI**	**Mean**	**95%CI**	**Mean**	**95%CI**	
Self-reported monthly household Income (in US$)	Before onset of TB symptoms	366	(297–434)	493	(81–904)	378	(305–451)	0.308
	At the time of TB diagnosis	317	(251–384)	252	(171–333)	311	(250–371)	0.532

Table S2. Body mass index at four time points of data collection, by study group

Variable	Category	Observation group N (%)	Intervention group N (%)	Total N (%)	p-value
**Total**		**154 (51.9%)**	**143 (48.1%)**	**297 (100%)**	-
**Nutritional status**					
Enrolment	Mean (SD)	19.6 (3.5)	19.9 (3.6)	19.7 (3.6)	0.521
	BMI: < 16.5	33 (21.4)	26 (18.2)	59 (19.9)	0.773
	BMI: 16.5-18.5	30 (19.5)	28 (19.6)	58 (19.5)	
	BMI: ≥ 18.5	91 (59.1)	89 (62.2)	180 (60.6)	
	BMI: < 18.5	63 (40.9)	54 (37.8)	117 (39.4)	0.663
	BMI: ≥ 18.5	91 (59.1)	89 (62.2)	180 (60.6)	
End of intensive phase	Mean (SD)	20.1 (3.4)	20.9 (3.5)	20.5 (3.5)	0.078
	BMI: < 16.5	20 (13.7)	11 (8.2)	31 (11.1)	0.164
	BMI: 16.5-18.5	27 (18.5)	19 (14.2)	46 (16.4)	
	BMI: ≥ 18.5	99 (67.8)	104 (77.6)	203 (72.6)	
	Unknown	8 (5.5)	9 (6.7)	17 (6.1)	
	BMI: < 18.5	47 (32.2)	30 (22.4)	77 (27.5)	0.089
	BMI: ≥ 18.5	99 (67.8)	104 (77.6)	203 (72.6)	
	Unknown	8 (5.5)	9 (6.7)	17 (6.1)	
Middle of continuation phase	Mean (SD)	20.9 (3.3)	21.4 (3.3)	21.2 (3.3)	0.190
	BMI: < 16.5	12 (8.4)	6 (4.6)	18 (6.6)	0.311
	BMI: 16.5-18.5	22 (15.4)	16 (12.3)	38 (13.9)	
	BMI: ≥ 18.5	109 (76.2)	108 (83.1)	217 (79.5)	
	Unknown	3 (2.1)	4 (3.1)	7 (2.6)	
	BMI: < 18.5	34 (23.8)	22 (16.9)	56 (20.5)	0.211
	BMI: ≥ 18.5	109 (76.2)	108 (83.1)	217 (79.5)	
	Unknown	3 (2.1)	4 (3.1)	7 (2.6)	
End of continuation phase	Mean (SD)	21.5 (3.5)	21.9 (3.2)	21.7 (3.3)	0.310
	BMI: < 16.5	8 (5.8)	4 (3.1)	12 (4.5)	0.211
	BMI: 16.5-18.5	19 (13.7)	11 (8.5)	30 (11.2)	
	BMI: ≥ 18.5	112 (80.6)	114 (88.4)	226 (84.3)	
	Unknown	4 (2.9)	1 (0.8)	5 (1.9)	
	BMI: < 18.5	27 (19.4)	15 (11.6)	42 (15.7)	0.113
	BMI: ≥ 18.5	112 (80.6)	114 (88.4)	226 (84.3)	
	Unknown	4 (2.9)	1 (0.8)	5 (1.9)	

Table S3. Results of linear mixed-effect model assessing BMI change over TB treatment phases, by study group

Effect	Estimate	Standard error	p-value
**Time Trend in Observation Group**			
Enrolment	19.619	0.278	<0.001
End of intensive phase	0.580	0.125	<0.001
Middle of continuation phase	1.313	0.126	<0.001
End of continuation phase	1.868	0.127	<0.001
**Main Effect of Intervention Group (at enrolment)**			
Study group: Intervention (vs Observation group)	0.266	0.400	0.507
**Difference in Time Trend: Intervention vs Observation**			
End of intensive phase (vs Intervention group)	0.252	0.181	0.165
Middle of continuation phase	-0.005	0.183	0.979
End of continuation phase	-0.103	0.184	0.576

Table S4. Factors associated with deaths due to TB

Age was again a significant factor for TB-related deaths, with individuals 65 and older having 5.68 times higher odds of death compared to those aged 0–44 ($p = 0.012$). HIV status remained a critical determinant, with HIV-positive patients facing 10.51 times higher odds of death than HIV-negative patients ($p < 0.001$). BMI at diagnosis showed a trend towards higher death rates in underweight patients, but this was not statistically significant in the adjusted analysis.

Variable		N	n (%)	p-value	Crude OR (95% CI, p-value)	Adjusted OR (95% CI, p-value)
Age group	0-44	121	9 (7.4)	0.546	Ref	Ref
	45-64	115	8 (7.0)		0.93 (0.34-2.52, p = 0.886)	2.18 (0.66-7.69, p = 0.205)
	65+	61	7 (11.5)		1.61 (0.55-4.56, p = 0.367)	5.68 (1.51-23.85, p = 0.012)
Sex	Female	112	4 (3.6)	0.046	Ref	-
	Male	185	20 (10.8)		3.27 (1.20-11.48, p = 0.035)	-
Marital status	Single	72	6 (8.3)	0.931	Ref	-
	Married	180	15 (8.3)		1.00 (0.39-2.90, p = 1.000)	-
	Divorced/separated /widowed	45	3 (6.7)		0.79 (0.16-3.15, p = 0.743)	-
Insurance type	With insurance	189	12 (6.3)	0.220	Ref	-
	No insurance	108	12 (11.1)		1.84 (0.79-4.30, p = 0.152)	-
Smoking status	No smoking experience	149	9 (6.0)	0.029	Ref	-
	Current smoker	29	6 (20.7)		4.06 (1.26-12.37, p = 0.015)	-
	Ex-smoker	119	9 (7.6)		1.27 (0.48-3.36, p = 0.621)	-
Alcohol use	Rarely/Never	212	15 (7.1)	0.384	Ref	-
	Monthly	29	2 (6.9)		0.97 (0.15-3.71, p = 0.972)	-
	Weekly	31	5 (16.1)		2.53 (0.77-7.15, p = 0.096)	-
	Daily	25	2 (8.0)		1.14 (0.17-4.40, p = 0.866)	-
HIV status	HIV negative	255	15 (5.9)	0.002	Ref	Ref
	HIV positive	41	9 (22.0)		4.50 (1.76-11.00, p = 0.001)	10.51 (3.17-38.04, p < 0.001)
	Status unknown	1	0 (0.0)		-	-
Educational level	Diploma or higher, vocational, other	57	6 (10.5)	0.476	Ref	-
	Lower/higher secondary	134	12 (9.0)		0.84 (0.31-2.51, p = 0.734)	-
	Primary	76	3 (3.9)		0.35 (0.07-1.39, p = 0.150)	-
	No education	30	3 (10.0)		0.94 (0.19-3.88, p = 0.939)	-
Employment status before TB	Unemployed	75	6 (8.0)	0.200	Ref	-
	Formal paid work	46	6 (13.0)		1.73 (0.51-5.86, p = 0.372)	-
	Informal paid work	141	12 (8.5)		1.07 (0.40-3.19, p = 0.897)	-
	Retired/student/housework/other	35	0 (0.0)		-	-
Nutritional intervention	Intervention group	143	12 (8.4)	1.000	Ref	-
	Observation group	154	12 (7.8)		0.92 (0.40-2.15, p = 0.850)	-
TB type	Pulmonary, bacteriologically confirmed	212	18 (8.5)	0.621	Ref	-

	Pulmonary, bacteriologically unconfirmed (clinically diagnosed)	69	4 (5.8)		0.66 (0.19-1.85, p = 0.472)	-
	Extrapulmonary	16	2 (12.5)		1.54 (0.23-6.11, p = 0.587)	-
Treatment history	New	281	23 (8.2)	1.000	Ref	-
	Relapse/retreatment	16	1 (6.2)		0.75 (0.04-3.96, p = 0.783)	-
Household size	< 5	148	9 (6.1)	0.295	Ref	-
	≥ 5	149	15 (10.1)		1.73 (0.74-4.24, p = 0.212)	-
Diagnostic delay	Yes	146	11 (7.5)	0.899	Ref	-
	No	151	13 (8.6)		1.16 (0.50-2.72, p = 0.734)	-
BMI at TB diagnosis	≥ 18.5	180	11 (6.1)	0.185	Ref	Ref
	< 18.5	117	13 (11.1)		1.92 (0.83-4.53, p = 0.128)	1.55 (0.61-3.94, p = 0.355)
